# Supplementary material for: The cryo-EM structure of ASK1 reveals an asymmetric architecture allosterically modulated by TRX1
Source: eLife. 2024 Mar 27;13:RP95199. doi: 10.7554/eLife.95199 (PMC10972558; doi:10.7554/eLife.95199)
Supplement: Supplementary file 1. [file elife-95199-supp1.docx]

**Supplementary File 1**

**Cryo-EM data collection, refinement and validation statistics**

| **Data collection and image processing** | |
| --- | --- |
| Instrument | FEI Titan Krios |
| Camera | K3 Gatan |
| Magnification | 105,000 |
| Voltage (kV) | 300 |
| Electron exposure (e^–^/Å^2^) | 40 |
| Defocus range (μm) | −0.7 to −2.8 |
| Pixel size (Å) | 0.834 |
| Total number of micrographs 0°tilt (no.) | 2704 |
| Total number of micrographs 40°tilt (no.) | 8691 |
| Selected micrographs (mixed, no.) | 5781 |
| Initial particle images | 1126189 |
| Final particle images (mixed, no.) | 645915 |
| Starting model | Ab initio |
| Symmetry | C1 |
| Map resolution, masked (Å)/FSC threshold | 3.71/0.143 |
| Sharpening | phenix.autosharpen |
| EMDB code | EMD-18396 |
|  |  |
| **Model building and refinement** | |
| Initial models used, PDB or AlphaFold Protein Structure database codes: | 2CLQ (KD)  5ULM (CRR)  AF-Q99683-F1 (TBD) |
| Masked FSC (map-model)/FSC threshold | 3.66/0.143 |
| Non-hydrogen atoms | 12744 |
| Amino acid residues | 1589 |
| Protein molecules | 2 |
| Real-space correlation - CCvolume | 0.64 |
| Real-space correlation - CCmasked | 0.64 |
| B factors (mean, Å^2^) | 97.92 |
| Root-mean-square deviation of bond length (Å)/angles (°) | 0.003/0.616 |
| PDB code | 8QGY |
|  | |
| **Validation** | |
| MolProbity score | 2.09 |
| Clashscore | 4.56 |
| Rotamer outliers (%) | 3.9 |
| Ramachandran plot - Favored (%)/Allowed (%)/Disallowed (%) | 93.77/6.17/0.06 |
